# Spontaneous hyperinflation of a giant bulla of the non-ventilated lung during laparoscopic cholecystectomy under one-lung ventilation: a case report

**DOI:** 10.1186/s40981-022-00552-1

**Published:** 2022-08-09

**Authors:** Taku Mayahara, Ryosuke Fukuoka, Norihiro Shimada, Junji Nishiyama

**Affiliations:** Department of Anesthesiology, Kobe Ekisaikai Hospital, Kobe, Japan

**Keywords:** Giant bulla, Hyperinflation, Laparoscopic surgery, One-lung ventilation

## Abstract

**Background:**

Anesthetic management of non-thoracic surgery in patients with giant bullae is challenging. We present a case of laparoscopic cholecystectomy in a patient with a giant bulla managed with one-lung ventilation (OLV).

**Case presentation:**

A 75-year-old man with a giant bulla occupying the lower half of the right hemithorax underwent laparoscopic cholecystectomy. We managed anesthesia with OLV to avoid positive pressure ventilation of the giant bulla. Surgery was completed uneventfully; however, postoperative chest radiography indicated a large lucency occupying the entire right hemithorax. Although we suspected a pneumothorax due to a ruptured bulla, chest computed tomography (CT) led to a diagnosis of giant bulla hyperinflation. The giant bulla deflated gradually to its preoperative size within three postoperative days.

**Conclusions:**

Managing laparoscopic cholecystectomy in a patient with a giant bulla with OLV resulted in spontaneous hyperinflation of the giant bulla. Chest CT ruled out a pneumothorax.

## Background

Anesthetic management of non-thoracic surgery in patients with giant bullae is challenging, and the main concern is to avoid positive pressure ventilation (PPV)-induced rupture of the bulla and perioperative tension pneumothorax [1, 2]. Regarding upper abdominal surgery, only one case report has described the successful anesthetic management of splenectomy in a patient with a giant bulla with one-lung ventilation (OLV) using a bronchial blocker [3]. Here, we present a case of laparoscopic cholecystectomy in a patient with a giant bulla managed with OLV using a double-lumen tube (DLT), which resulted in hyperinflation of the giant bulla of the non-ventilated lung.

## Case presentation

**Fig. 1:**
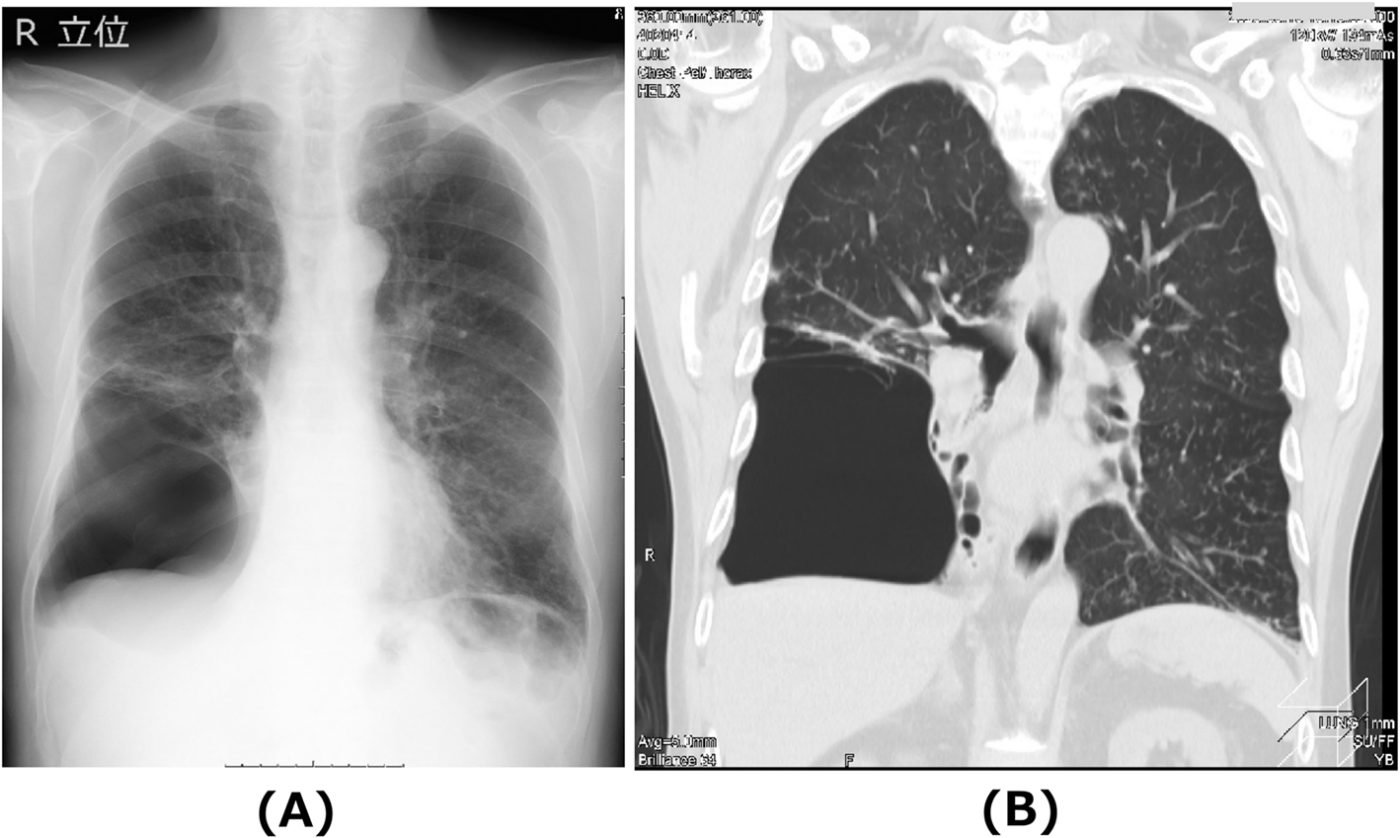
Preoperative chest radiography (**A**) and computed tomography (**B**) images. The giant bulla occupying the lower half of the right hemithorax compressed the middle and lower lobes of the right lung, leading to atelectasis. The right upper lobe had good air content

**Fig. 2:**
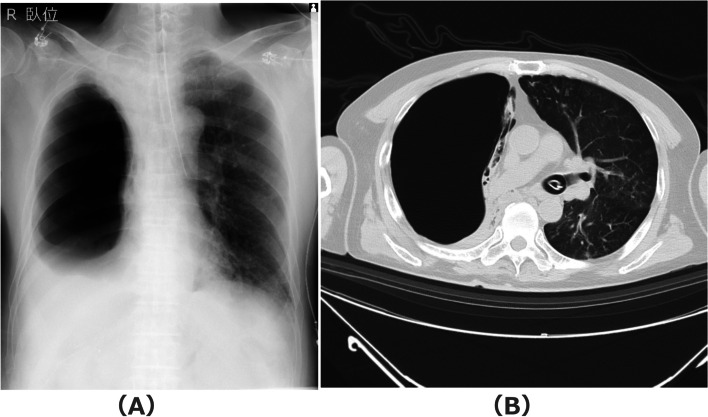
Postoperative chest radiography (**A**) and computed tomography (**B**) before extubation. Hyperinflated giant bulla compressed the whole right lung, including the upper lobe, to atelectasis

A 75-year-old man with acute cholecystitis was scheduled to undergo a laparoscopic cholecystectomy. He had a history of emphysema and spontaneous pneumothorax with no limitations in his daily activities. Preoperative respiratory function tests indicated mild obstructive disorder. Preoperative chest radiography indicated a giant bulla occupying the lower half of the right hemithorax (Fig. 1A). Computed tomography (CT) of the chest indicated that the giant bulla compressed the middle and lower lobes of the right lung, leading to atelectasis. Conversely, the upper lobe had good air content (Fig. 1B). To prevent rupture of the giant bulla and tension pneumothorax during laparoscopic cholecystectomy, we planned to manage the patient under general anesthesia with OLV. Anesthesia was induced with propofol 80 mg, fentanyl 100 mcg, rocuronium 60mg, and maintained with 4–5% desflurane and remifentanil 0.13–0.18 mcg/kg/min. After two to three times of manual ventilation via a facemask, the patient’s trachea was intubated with a 37-Fr left DLT. OLV was started immediately after intubation and continued throughout the surgery, with the right-sided lumen open to ambient air. The surgery was completed uneventfully in 125 min; peripheral oxygen saturation was 98–99% throughout the surgery. Chest radiography before extubation indicated a large lucency occupying the entire right hemithorax (Fig. 2A). Initially, we suspected a pneumothorax due to rupture of the giant bulla; however, chest tube insertion was withheld because his hemodynamics and respiratory status were stable. Instead, we continued the OLV and obtained a chest CT scan to determine whether any conditions other than pneumothorax were present. Chest CT (Fig. 2B) indicated compression atelectasis of the entire right lung, including the upper lobe wrapped around the giant bulla. A small amount of pleural fluid did not form a liquid surface. Instead, it was compressed by a giant bulla to form a concave surface. These findings led us to diagnose hyperinflation of the giant bulla rather than a pneumothorax. Returning to the operating room, an attempt was made to relieve atelectasis using a recruitment maneuver with moderate pressure. However, follow-up radiography indicated that the right lung had not expanded. We decided to observe the hyperinflated giant bulla conservatively under spontaneous breathing and extubated the patient after emergence from general anesthesia. After extubation, the patient’s respiratory status was normal with no evident abnormalities. The hyperinflated giant bulla deflated and the right upper lobe gradually expanded under spontaneous breathing. The chest radiograph on postoperative day three was similar to the preoperative radiograph. The patient was discharged on postoperative day seven.

## Discussion

Bullous emphysema is typically observed in patients with chronic obstructive pulmonary disease, and when a bulla occupies more than 30% of the hemithorax, it is called a giant bulla [4]. Non-thoracic surgery in patients with giant bullae is rare, and only a few case reports have described anesthetic management. When managing patients with giant bullae, the main anesthetic concern is avoiding PPV-induced rupture of the bulla and perioperative tension pneumothorax. Therefore, anesthetic management under spontaneous breathing is preferred for surgeries in which PPV is not essential, such as surgery of the extremities and lower abdomen [1, 2]. However, upper abdominal surgery usually requires general anesthesia and PPV to provide patient comfort and an excellent surgical field. Only one case report has described the successful anesthetic management of splenectomy with OLV using a bronchial blocker in a patient with a giant bulla [3]. Because laparoscopic cholecystectomy is an upper abdominal surgery and pneumoperitoneum requires precise respiratory control, we decided to manage the patient with general anesthesia and OLV using a DLT. Consequently, general anesthesia with OLV successfully prevented giant bulla rupture in our patient. However, we unexpectedly found hyperinflation of the giant bulla and compression atelectasis of the entire right lung at the end of surgery.

As is often observed in video-assisted thoracic surgery, healthy lungs quickly collapse when the pleura is opened, and ambient air freely enters the thoracic cavity [5]. Conversely, emphysematous lungs and bullae are reluctant to collapse because of reduced elastic recoil and expiratory limitation [6]. Although the pleura was not open in our case, the right lumen of the DLT was open to ambient air throughout the OLV. It is likely that the open right-sided lumen of the DLT allowed ambient air to flow into the giant bulla, thereby enabling the right upper lobe to collapse.

Several measures could have been taken to prevent right upper lobe atelectasis and giant bulla hyperinflation during general anesthesia and OLV. Providing an OLV with a closed right-sided lumen may be the most straightforward option, although its effectiveness remains uncertain. It may be more effective if a Jackson-Rees circuit or another ventilator is connected to the right lumen of the DLT to apply a mild positive end-expiratory pressure to the right lung. Intubating a single-lumen tube and placing a bronchial blocker in the right intermediate bronchus may enable ventilation of the right upper lobe and left lung while preventing PPV of the giant bulla. Although laparoscopic cholecystectomy is typically performed under general anesthesia and PPV, recent studies have shown that it can be performed safely under neuraxial block and spontaneous breathing [7, 8]. Spinal anesthesia or combined spinal and epidural anesthesia with spontaneous breathing may have been a good alternative in our case.

Differentiating the hyperinflated giant bulla from pneumothorax was challenging on chest radiography. Generally, a giant bulla sometimes mimics a tension pneumothorax, and some case reports have described unnecessary chest tube insertion in patients with giant bullae [9, 10]. In such cases, CT is required to avoid misdiagnosis. On CT imaging, the lung collapses toward the ipsilateral hilum in the case of pneumothorax, whereas it is draped around the bulla in the case of a giant bulla [4, 9]. CT imaging effectively differentiated the hyperinflated giant bulla from pneumothorax in our case and enabled us to avoid unnecessary chest tube insertion.

## Conclusions

We managed a case of laparoscopic cholecystectomy with OLV using a DLT in a patient with a giant bulla, which resulted in hyperinflation of the giant bulla. CT imaging helped differentiate between hyperinflation of the giant bulla and pneumothorax.

## Data Availability

Not applicable.

## Abbreviations

PPV: Positive pressure ventilation
OLV: One-lung ventilation
DLT: Double-lumen tube
CT: Computed tomography
